# The comparative morphology of the oral cavity glands in captive South African painted dogs (*Lycaon pictus pictus*) and captive fennec foxes (*Vulpes zerda*) (Carnivora: Canidae)

**DOI:** 10.1186/s12917-024-04305-3

**Published:** 2024-10-10

**Authors:** Joanna Klećkowska-Nawrot, Krzysztof Stegmann, Arkadiusz Dziech, Gabriela Jędrszczyk, Igor Jucenco, Karolina Barszcz, Karolina Goździewska-Harłajczuk

**Affiliations:** 1 Department of Biostructure and Animal Physiology, Faculty of Veterinary Medicine, Wrocław University of Environmental and Life Sciences, Kozuchowska 1, Wrocław, 51-631 Poland; 2 GeoWild, Marsz. Jozefa Pilsudskiego 74 lok. 320, Wrocław, 50-020 Poland; 3 Department of Genetics, Genetics of Populations and Quantitative Traits, Wrocław University of Environmental and Life Sciences, Kozuchowska 7, Wrocław, 51-631 Poland; 4 Faculty of Veterinary Medicine, Student Anatomical Club “Alkmeon”, Wrocław University of Environmental and Life Sciences, Norwida 25, Wrocław, 50-375 Poland; 5 Department of Morphological Sciences, Institute of Veterinary Medicine, Warsaw University of Life Sciences, Nowoursynowska 159, Warsaw, 02-787 Poland

**Keywords:** Anatomy, Histology, Histochemistry, Zygomatic gland, Monostomatic sublingual gland, Polystomatic sublingual gland, Mandibular gland, Parotid gland

## Abstract

**Background:**

The African painted dog is classified as a relic canid lineage, inhabiting areas south of the Sahara. The fennec fox is the smallest member of the Canidae family, found in the Arabian Peninsula and northern Sahara.

**Methods:**

The gross anatomy and light microscopic examination of the oral cavity glands were studied in five adult captive South African painted dogs and five adult captive fennec foxes from the Wroclaw Zoological Garden, Poland. In this research, the zygomatic gland, monostomatic sublingual gland, polystomatic sublingual gland, mandibular gland, and parotid gland were examined for their topography, morphometry, histology, and histochemistry using hematoxylin and eosin, azan trichrome, mucicarmine, PAS, AB pH 1.0, AB pH 2.5, AB pH 2.5 PAS, and HID.

**Results:**

We found that the parotid glands were consistently the largest, followed by the mandibular and sublingual glands (both monostomatic and polystomatic). The zygomatic gland was the smallest in both South African painted dogs and fennec foxes. Interestingly, there were noticeable differences in the size, shape, and even composition of the secretory products between the two species. The zygomatic and polystomatic sublingual glands in the South African painted dog and the fennec fox were complex branched tubular. In the South African painted dog, the monostomatic gland was a branched tubular compound gland, while in the fennec fox, it was a branched tubuloalveolar compound gland. The mandibular gland in hunting dogs was a branched tubular compound gland, while in the fennec fox a branched tubuloalveolar compound gland. The parotid gland in the fennec fox was a branched acinar compound gland, whereas in the painted dog was a branched tubuloacinar.

**Conclusions:**

The basic structure of their glands is similar to that of other terrestrial carnivores, indicating a shared evolutionary origin and function. However, differences in the composition of their secretory products can reflect adaptations to their specific diets. This research provides valuable insights for veterinary medicine and underscores the importance of further studies. By analyzing wild canid populations and including a broader range of species with diverse diets, we could gain a deeper understanding of how diet influences salivary gland morphology within the Canidae.

## Background

The South African painted dog (*Lycaon pictus pictus*), also known as painted dog or Cape hunting dog, inhabits sub-Saharan Africa, primarily in East and Southern Africa, with scattered populations in the western and central Sahel countries [1, 2]. The painted dog is a carnivore, with its most common prey species being medium-sized antelopes weighing between 15 and 45 kg, such as impala (*Aepyceros melampus*), Nyala (*Tragelaphus angasii*), lechwe (*Kobus leche*), the Cephalophinae subfamily, bushbuck (*Tragelaphus scriptus*), and Thomson’s gazelle (*Eudorcas thomsonii*) [2–4]. They also hunt larger prey, such as greater kudu (*Tragelaphus strepsiceros*), zebras, African buffalo (*Syncerus caffer*), common wildebeest (*Connochaetes taurinus*), or desert warthog (*Phacochoerus aethiopicus*) [2, 5, 6], while including smaller-sized prey like Kirk’s dik-dik (*Madoqua kirkii*) or steenbok (*Raphicerus campestris*) in their diet [2, 7, 8]. MacDonald and Sillero-Zubiri [4] reported that the diet of the Cape hunting dog varies significantly depending on the availability of different ungulate species and warthogs.

The fennec fox (*Vulpes zerda*), hereafter also referred as fox, is distributed across North Africa, ranging from the western Sahara Desert and Morocco to the Sinai Peninsula, and extending to the Nubian Desert on the Red Sea [1, 9]. The fennec fox is an omnivore that feeds on a variety of small species, mainly rodents such as the lesser Egyptian jerboa (*Jaculus jaculus*), Sundevall’s jird (*Meriones crassus*), Western Mediterranean mouse (*Mus spretus*), and representatives of the Gerbillidae family. It also consumes lizards, geckos, skinks, small birds and their eggs, invertebrates (most common being Coleoptera and Isoptera) and their larvae, as well as fruits, leaves, roots, and some tubers [2, 10, 11]. The diet varies across different regions and likely depends on the availability and abundance of prey; for example, in some regions, mammals make up the most significant portion of the diet (biomass), while in others, plant material is the primary source of food [10].

The *Nomina Anatomica Veterinaria* (*NAV*) [12] classifies the glands of the oral cavity into minor and major salivary glands to provide a standardized anatomical terminology for veterinary medicine. Minor salivary glands, including the labial, buccal, molar, palatine, lingual, and paracaruncular glands, are small clusters present in the mucous membranes of the oral cavity [12–14]. The zygomatic gland, although classified as a minor salivary gland, is characterized by its significant size and has one major duct that opens at the zygomatic papilla and 2 to 4 minor ducts that open into the oral vestibule [12]. The major salivary glands (monostomatic sublingual gland, polystomatic sublingual gland, mandibular gland, and parotid gland) provide saliva to the oral cavity, including the vestibule and the oral cavity proper, through simple excretory ducts [12–14].

The composition of saliva in mammals varies depending on multiple factors, such as age, sex, health, environmental conditions (time of day and year), physical activity, diet and the type of stimulation that triggers saliva production (mechanical, chemical, or psychoneurological) [15]. Saliva plays a crucial role in digestion, lubrication, waste removal, and taste perception. Differentiation in composition is also visible between different individuals of the same species [16–18]. These factors, in addition to influencing saliva composition, have an impact on the size of the oral cavity glands, their architecture, and the nature of the secretion produced (serous, mucous, or mixed – mucoserous or seromucous) [16–19].

The morphology of salivary glands of the Canidae species were described in detail for a domestic dog (*Canis lupus familiaris*) [20–36]. Furthermore, anatomical and histological research of these glands has been described for the red fox (*Vulpes vulpes*) [37], crab-eating fox (*Cerdocyon thous*) [38], and pampas fox (*Lycalopex gymnocercus*) [39].

The aim of the study was to determine whether the diet of our animals, predominantly consisting of animal-derived products (such as meat, insects), influences differences in the morphology of the salivary glands. We compared our findings with those of other terrestrial carnivores with diverse diets, based on the available literature. Addiitionally, the study aimed to understand the morphology of the salivary glands, which is crucial for veterinary medicine, particularly in facilitating the diagnosis of diseases affecting these anatomical structures. The disorders of the salivary gland, such as primary and secondary tumors, nonspecific sialadenitis, salivary cysts, and necrotizing sialometaplasia [40–43], can significantly impact the health and well-being of animals, especially wild canids that inhabit zoological gardens or national parks. A thorough understanding of the morphology of these glands is essential for the accurate diagnosis and treatment of these conditions, which can directly affect strategies for managing the health and conservation of wild animal populations.

## Materials and methods

### Animals

The study was carried out on five adult South African painted dogs (each with a mean weight of around 20–25 kg) and five adult fennec foxes (two males and three females) (with each male weighing approximately 1.40–1.50 kg and each female weighing approximately 0.8–0.9 kg). The study samples were collected from 2016 to 2024. All animals were obtained from the Wrocław Zoological Garden in Poland. These animals were collected after their natural deaths, and no animals were killed specifically for the purpose of this study. The entire research material was collected in the Division of Animal Anatomy at Wrocław University of Environmental and Life Sciences.

## Anatomical dissection

Immediately after the animals’ death (*n* = 10), the salivary glands (zygomatic gland, monostomatic sublingual gland, polystomatic sublingual gland, mandibular gland, and parotid gland) were dissected for macroscopic measurements and morphological description. Measurements of the examined glands were conducted using a digital caliper with a resolution of 0.01 mm and an accuracy of ± 0.02 mm (> 100 mm) (Handy Worth, Poland). The values for six measurements (length, width and thickness) were recorded as mean ± standard deviation. The morphological description of these salivary glands was based on topographic anatomy methods [44]. Nomenclature from the *Nomina Anatomica Veterinaria* [12] and Weterynaryjne Mianownictwo Anatomiczne (Lat-Pol-Eng) [45] was used for the description of the studied salivary glands.

Subsequently, salivary gland samples were taken for light microscopy, measuring 0.5 cm x 0.5 cm, and placed in plastic containers filled with 4% buffered formaldehyde. The entire animals were then placed in plastic tanks and preserved in a 20% formalin solution for 5 days. Macroscopic pictures were captured with a Nikon D300s camera equipped with a Tamron AF 17–50 mm F/2.8 [IF] ∅ 67 lens.

## Histological and histochemical study

The gland samples collected were fixed in 4% buffered formaldehyde for 48 h. They were then rinsed in running water for 24 h and dehydrated using solutions of 75%, 96%, and 100% ethanol. The paraffin blocks were sliced with a Micron HM310 microtome into 5 μm sections. Staining methods including hematoxylin and eosin, azan trichrome, mucicarmine, periodic acid-Schiff (PAS), alcian blue pH 1.0 (AB pH 1.0), alcian blue pH 2.5 (AB pH 2.5), AB pH 2.5/PAS, and high iron diamine (HID) were applied [46, 47]. Histochemical studies were performed to evaluate the composition of secretory units and were methods described by Spicer and Henson [47] as follows: (–) indicated a negative reaction; (–/+) and (+) a weak positive reaction; (++) and (++/+++) a moderate positive reaction; and (+++) a strong positive reaction. The slides were analyzed using a Zeiss Axio Scope A1 light microscope (Carl Zeiss, Jena, Germany) and histological descriptions based on the *Nomina Histologica Veterinaria* [48].

## Results

The largest salivary glands in the studied hunting dog were the parotid glands, followed by the mandibular glands, sublingual glands, which included both monostomatic and polystomatic types (Table 1). Finally, the zygomatic glands were the smallest. Meanwhile, in the fennec fox, the largest was the parotid gland, and the mandibular, monostomatic, and polystomatic sublingual glands were very similar in size (Table 1). The smallest gland in this species was the zygomatic gland. The longest excretory duct in all animals examined was associated with the mandibular gland, followed by the parotid glands and the major sublingual gland. The major sublingual duct was longer in male fennec foxes compared to females (Table 1).

**Table 1:** Morphometry (mm) of the zygomatic gland, major salivary glands and ducts in the captive South African painted dog (*Lycaon pictus pictus*) (*n =* 5 female) and captive fennec fox (*Vulpes zerda*) (*n =* 2 male, *n* = 3 female). Mean ± S.D

Measurement	Zygomatic gland			Monostomatic sublingual gland			Major sublingual duct	Polystomatic sublingual gland			Mandibular gland			Mandibular duct	Parotid gland			Parotid duct
	length	width	thickness	length	width	thickness	length	length	width	thickness	length	width	thickness	length	length	width	thickness	length
**Female South African painted dog**	19.043 ± 0.8	11.581 ± 0.5	5.977 ± 0.2	31.307 ± 0.6	8.916 ± 0.2	3.74 ± 0.3	49.656 ± 0.8	26.31 ± 1.2	6.211 ± 0.4	3.991 ± 0.5	46.51 ± 1.0	16.253 ± 0.5	17,309 ± 0.6	81.114 ± 1.2	58.515 ± 1.2 56.775 ± 1.0 40.795 ± 1.1 25.440 ± 1.1	14.224 ± 1.2 16.115 ± 0.7 10.898 ± 0.7 12.332 ± 1.1	6.712 ± 0.6 7.74 ± 0.4 5.897 ± 1.0 6.389 ± 0.5	71.613 ± 1.6
**Male** **fennec fox**	6.166 ± 0.6	5.425 ± 0.3	3.051 ± 0.3	13.304 ± 0.4	6.379 ± 0.7	2.602 ± 0.5	24.278 ± 0.9	15.865 ± 0.4	5.979 ± 0.4	2.81 ± 0.5	14.519 ± 0.8	12.896 ± 0.8	6.435 ± 0.6	41.969 ± 0.9	20.663 ± 0.01 13.208 ± 1.3 16.375 ± 1.0	8.437 ± 0.6 6.132 ± 0.4 11.642 ± 0.8	1.225 ± 0.3 2.065 ± 0.2 2.476 ± 0.4	31.341 ± 1.0
**Female** **fennec fox**	5.635 ± 0.5	4.931 ± 0.3	2.294 ± 0.3	12.924 ± 0.6	5.025 ± 0.5	2.805 ± 0.3	20.555 ± 0.7	15.123 ± 0.7	5.207 ± 0.5	2.776 ± 0.3	12.532 ± 0.5	12.390 ± 0.5	5.893 ± 0.3	39.416 ± 0.9	18.150 ± 0.9 13.239 ± 0.5 16.422 ± 0.4	7.392 ± 0.5 5.583 ± 0.5 10.466 ± 0.6	1.077 ± 0.2 1.97 ± 0.1 1.926 ± 0.1	30.262 ± 0.5

### The zygomatic gland

**Fig. 1:**
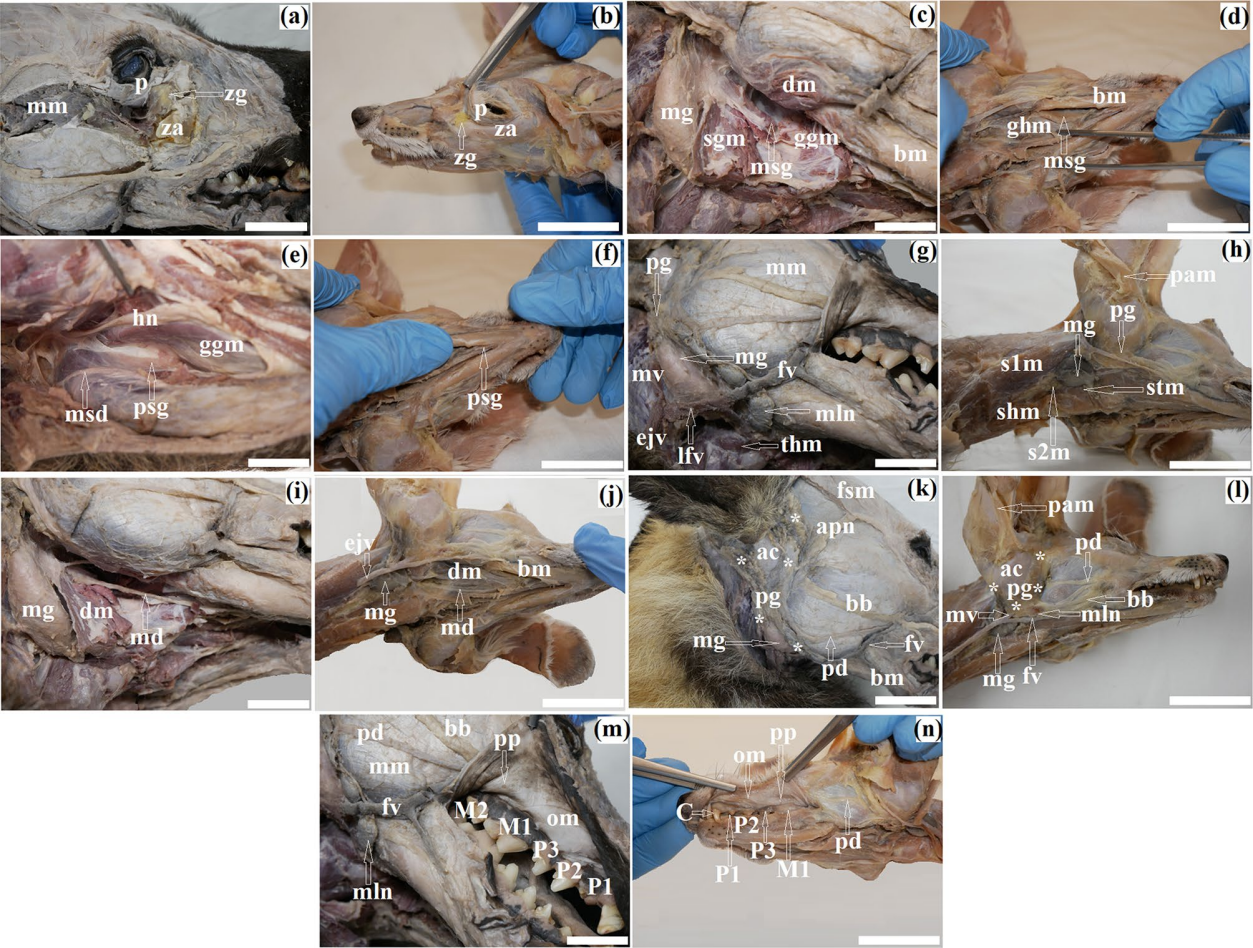
Gross anatomy images of the zygomatic gland and major salivary glands in the captive South African painted dog (**a**, **c**, **e**, **g**, **i**, **k**, **m**) and the captive fennec fox (**b**, **d**, **f**, **h**, **j**, **l**, **n**). ac – anular cartilage (external acustic meatus), apn – aurliculopalpebral nerve, bb – buccal branch (facial nerve), bm – body of mandible, C – Canine teeth, dm – digastrics muscle, ejv – external jugular vein, fsm – frontoscutular muscle, fv – facial vein, ggm – genioglossus muscle, ghm – geniohyoid muscle, hn – hypoglossal nerve, lfv – linguofacial vein, M (1, 2) – Molar teeth, md – mandibular duct, mg – mandibular gland, mln – mandibular lymph nodes, mm – masseter muscle, msd – major sublingual duct, msg – monostomatic sublingual gland, mv – maxillary vein, om – oral mucosa, P (1, 2, 3) – Premolar teeth, p – periorbita, pam – parotidoauricularis muscle, pd – parotid duct, pg – parotid gland (white asterisk), pp – parotid papilla, psg – polystomatic sublingual gland, s1m – sternocephalicus muscle (occipital part), s2m – sternocephalicus muscle (mastoid part), sgm – styloglossus muscle, shm – sternohyoid muscle, stm – sternothyroid muscle, thm – thyrohyoid muscle, za – zygomatic arch, zg – zygomatic gland. Bar: a – *n* = 2 cm

The zygomatic gland in the South African painted dog was located in the region of the zygomatic arch (Fig. 1a). Laterally, it abutted the masseter muscle and temporal muscle, while medially it bordered the periorbita, medial pterygoid muscle, maxillary artery, deep facia artery, and maxillary nerve. In contrast, in the fennec fox, this gland was located in the nasal part of the zygomatic arch, near the pterygopalatina fossa, at the border between the lacrimal and zygomatic facial parts (Fig. 1b). In the hunting dog, it had a slightly yellowish color, whereas in the fox, it was intensely yellow. In both examined species, it formed irregular clusters of small packets surrounded by a thick connective tissue capsule. It had 5 to 6 efferent ducts in the South African painted dog and 3 to 4 efferent ducts (1 major zygomatic duct and minor zygomatic ducts) which opened into the vestibule of the oral cavity behind the last upper molar (M2).

**Fig. 2:**
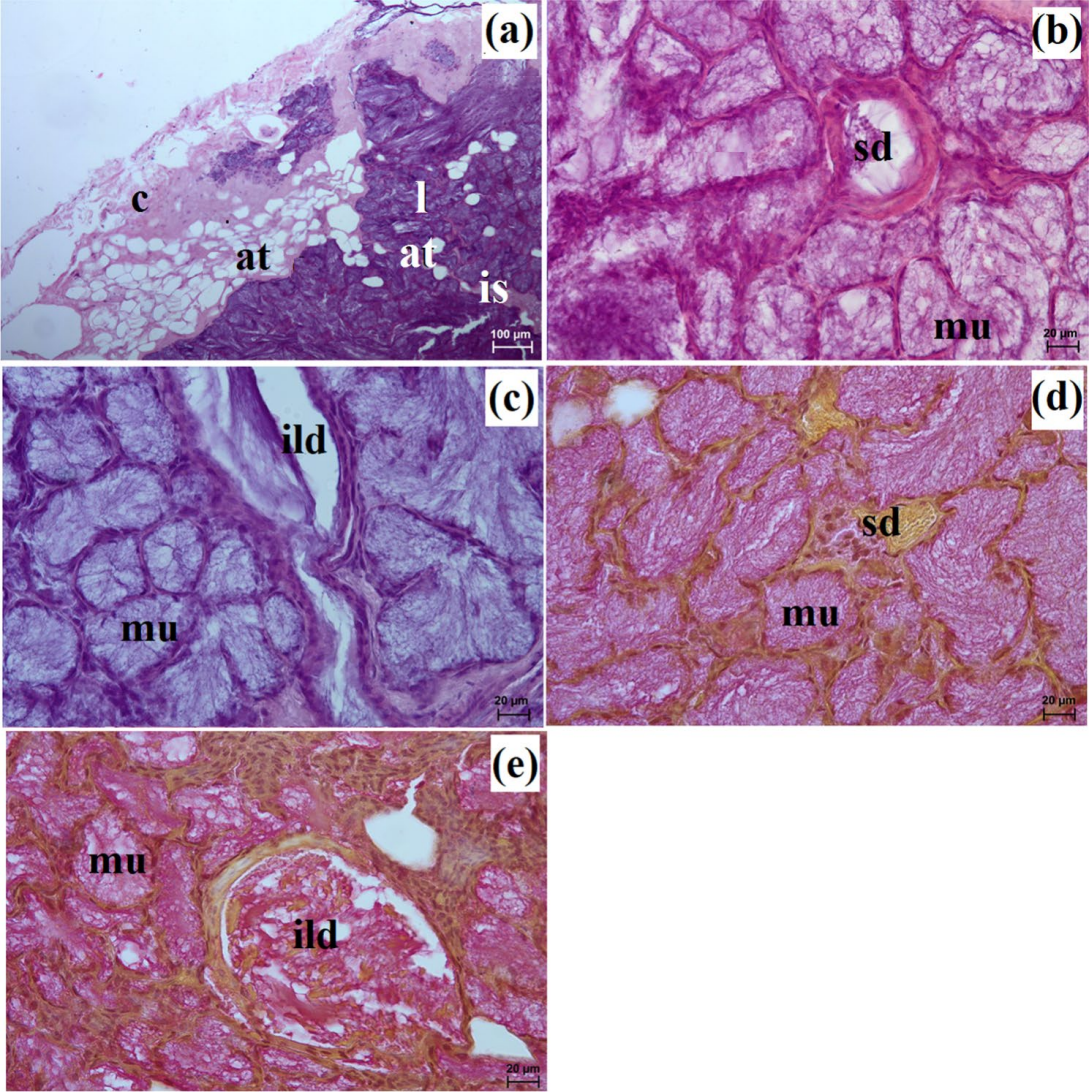
Histology images of the zygomatic gland in the captive South African painted dog. at – adipose tissue, c – capsule, ild – intralobular duct, is – interlobar septa, l – lobes, mu – mucous units, sd – striated duct. a – c = H&E stain; d, e = mucicarmine stain

**Fig. 3:**
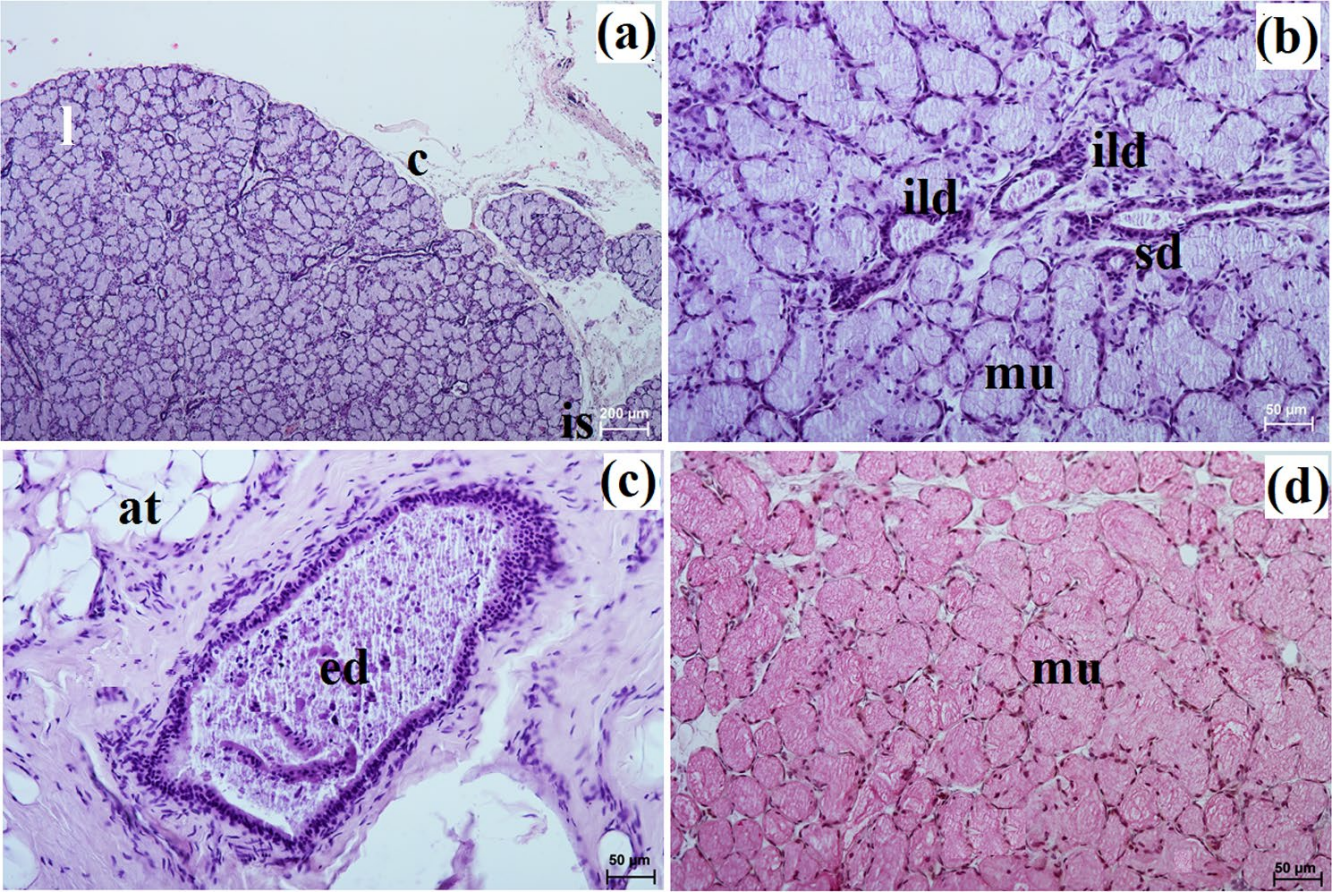
Histology images of the zygomatic gland in a captive fennec fox. at – adipose tissue, c – capsule, ed – excretory duct, ild – intralobular duct, is – interlobar septa, l – lobes, mu – mucous units, sd – striated duct. a – c = H&E stain; d = mucicarmine stain

**Fig. 4:**
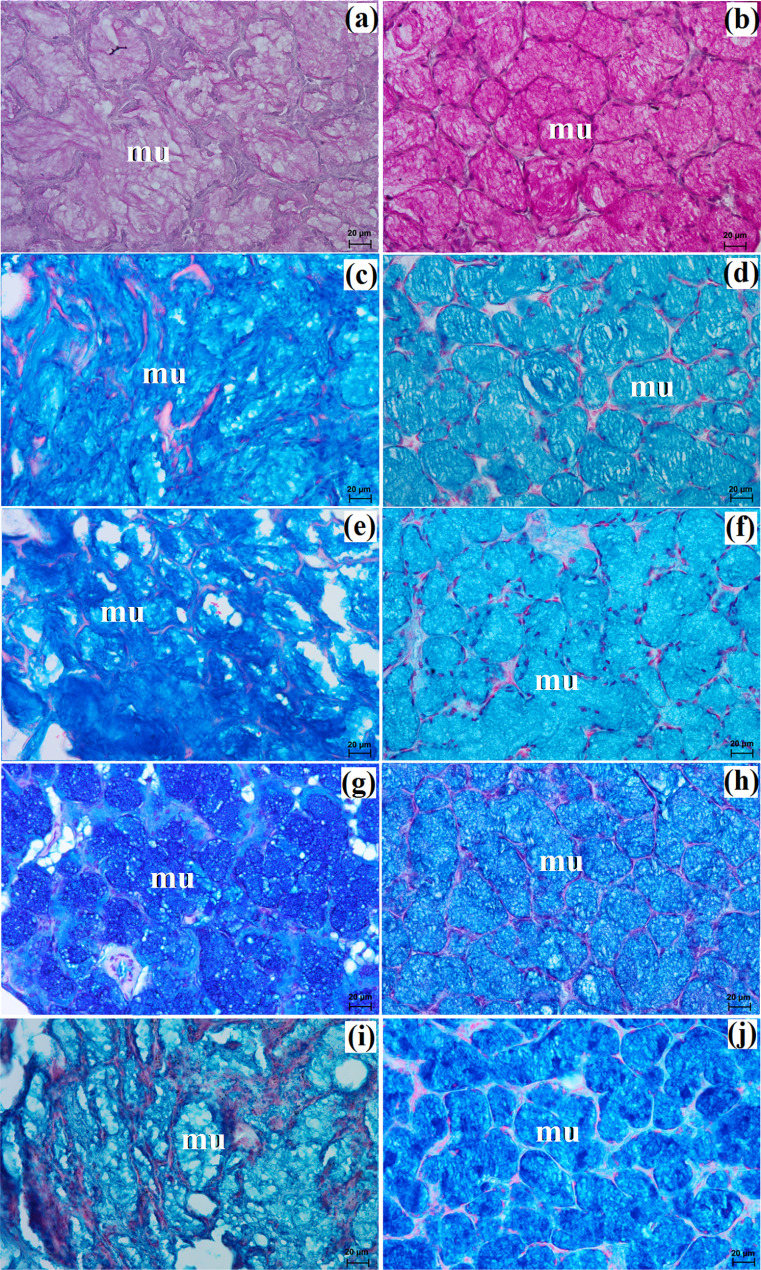
Histochemical images of the zygomatic gland in the captive South African painted dog (**a**, **c**, **e**, **g**, **i**) and the captive fennec fox (**b**, **d**, **f**, **h**, **j**). mu – mucous units. **a**, **b** = PAS; **c**, **da** = AB pH 1.0; **e**, **f** = AB pH 2.5; **g**, **h** = AB pH 2.5/PAS; **i**, **j** = HID

The zygomatic gland in all examined animals was surrounded by a thick connective tissue capsule (Figs. 2a and 3a), within which numerous clusters of adipose cells were found only in the South African painted dog (Fig. 2a). In a hunting dog, his gland was divided by interlobar septa, varying in thickness, into large oval lobes (Fig. 2a). Additionally, the connective tissue surrounding the lobes contained numerous clusters of adipose cells, which were also present between secretory units (Fig. 2a). In the fennec fox, thin septa divided the gland parenchyma into dominant large lobes and few small lobes, also with an oval shape (Fig. 3a). The duct system in the examined animals was well-developed (Figs. 2b and c and 3b). Intralobular ducts were composed of tall columnar cells with oval nuclei located at the basal membrane, while the striated ducts were composed of a simple cuboidal epithelium with nuclei located apically and basal striations (Figs. 2b and c and 3b). The excretory duct was composed of simple columnar epithelium (Fig. 3c). The zygomatic gland in all examined Canidae was complex branched tubular and produced mucous secretion (Fig. 4a and j; Table 2). The tubules were composed of low pyramidal cells with a broad base and flattened nuclei located at the basal part of the cell (Figs. 2c and 3b). Mucicarmine staining showed a strong (+++) positive reaction in mucous cells (Figs. 2d and e and 3d).

**Table 2:** Comparative histochemical characterization of the zygomatic gland and major salivary glands in captive South African painted dog (*Lycaon pictus pictus*) and captive fennec fox (*Vulpes zerda*). PAS – periodic acid–Schiff; AB pH 1.0 – alcian blue pH 1.0; AB pH 2.5 – alcian blue pH 2.5; HID – high iron diamine. (–) a negative reaction; (+) a weak positive reaction; (++) and (++/+++) a moderate positive reaction; and (+++) a strong positive reaction (Spicer and Henson, 1967)

**Histochemical stain**	Zygomatic gland		Monostomatic sublingual gland		Polystomatic sublingual gland		Mandibular gland		Parotid gland	
	South African painted dog	**Fennec** fox	South African painted dog	**Fennec** fox	South African painted dog	Fennec fox	South African painted dog	Fennec fox	South African painted dog	Fennec fox
PAS	(–)	(+++)	(+++)	(+) serous demilunes and (–) tubules	(+)	(++)	(+)	(–)	(+) serous demilunes and (+) tubules	(+)
**AB pH 1.0**	(+++)		(+++)	(+) serous demilunes and (+++) tubules	(+++)		(+++)	(–) serous demilunes and (+++) tubules	(–) serous demilunes and (+++) tubules	(–)
**AB pH 2.5**	(+++)		(+++)	(+) serous demilunes and (+++) tubules	(+++)		(+++)	(–) serous demilunes and (+++) tubules	(–) serous demilunes and (+++) tubules	(++)
**AB pH 2.5/PAS**	(+++) (blue color)	(++) (blue color)	(+++) (blue color)	(++) serous demilunes (magenta color) and (+++) tubules (blue color)	(+++) (blue color)		(+++) (blue color)	(+) serous demilunes (magenta color) and (+++) tubules (blue color)	(++) serous demilunes (magenta color) and (+++) tubules (blue color)	(+) (blue color)
**HID**	(+++)		(++)	(+) serous demilunes and (+++) tubules	(+++)	**(+++)**	(+++)	(–) serous demilunes and (+++) tubules	(+) serous demilunes and (+++) tubules	(++)

### The monostomatic sublingual gland

The monostomatic sublingual gland was located beneath the mucous membrane of the lateral sublingual recess of the floor of the oral cavity. It resided between the digastric muscle, styloglossus muscle, body of mandible, and pterygoid muscle. The posterior part of this gland abutted the anterior edge of the mandibular gland, while the anterior part made contact with the genioglossus muscle (Fig. 1c and d). The shape of this salivary gland in both examined species was elongated. The sublingual major duct ran together with the mandibular duct and opened into the proper oral cavity at the sublingual caruncle.

**Fig. 5:**
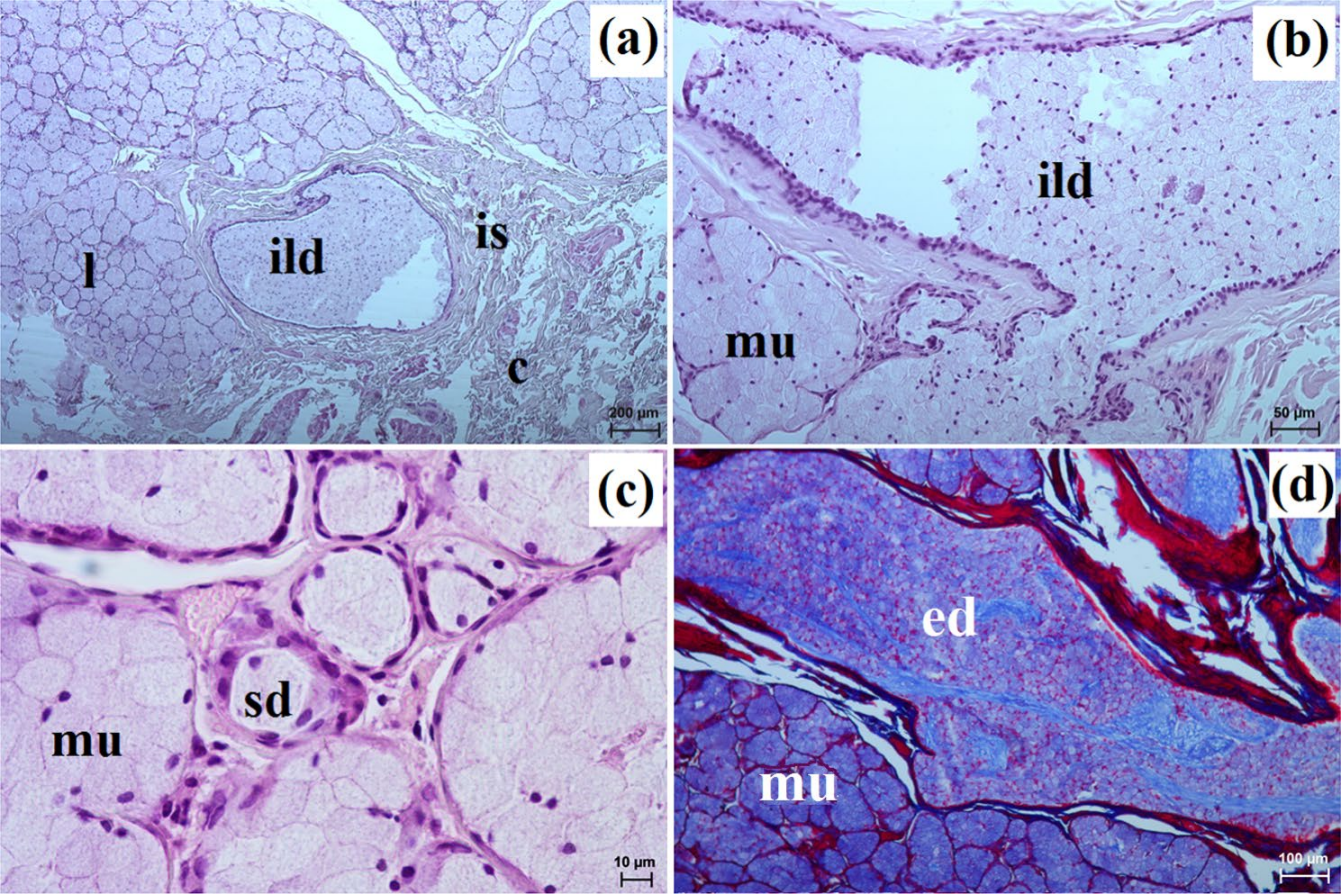
Histology images of the monostomatic sublingual gland in the captive South African painted dog. c – capsule, ed – excretory ducts, ild – intralobular duct, is – interlobar septa, l – lobes, mu – mucous units, sd – striated duct. **a** – **c** = H&E stain; **d** = azan trichrome stain

**Fig. 6:**
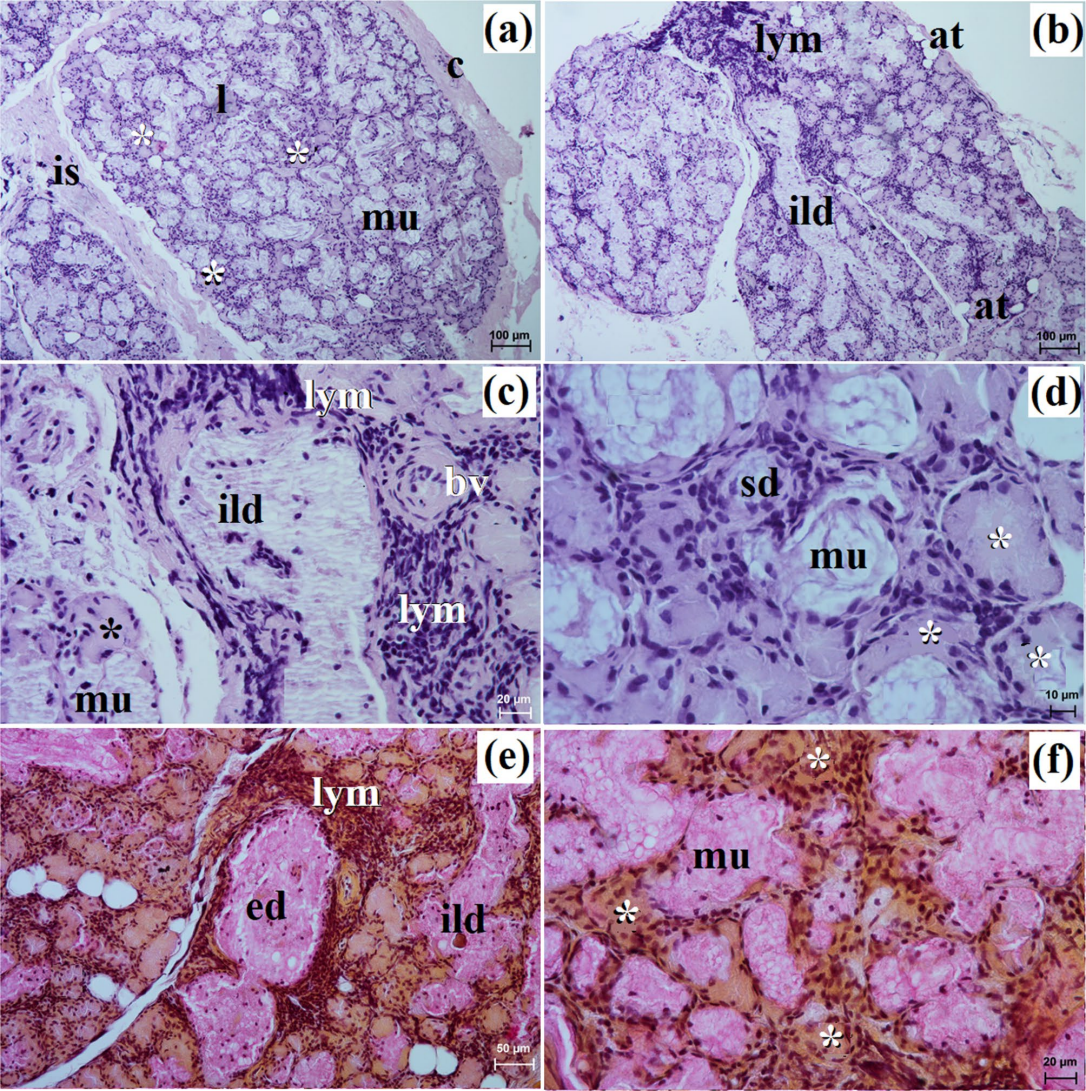
Histology images of the monostomatic sublingual gland in the captive fennec fox. at – adipose tissue, bv – blood vessels, c – capsule, ed – excretory duct, ild – intralobular duct, is – interlobar septa, l – lobes, lym – lymphocytes, mu – mucous units, sd – striated duct, white asterisk – serous acini. **a** – **d** = H&E stain; **e**, **f** = mucicarmine stain

This gland in all examined species was surrounded by a thick connective tissue capsule (Figs. 5a and 6a). In the South African painted dog, it was divided into numerous lobes of various sizes (small, medium, large), often elongated in shape due to thick interlobular septa. In the fennec fox, the thick and thin septa divided the gland parenchyma into dominant large lobes and few small lobes of oval shape (Fig. 6a). In the fennec fox, clusters of adipose cells were observed on the interlobular septa and within the gland stroma (Fig. 6b). In both, painted dog and fox, the striated ducts were composed of similar to those in the zygomatic gland (Figs. 5b and c and 6b and c). The intralobular ducts in all examined carnivores were composed of tall columnar cells with large oval nuclei. In the South African painted dog and fennec fox, excretory ducts were present (Figs. 5d and 6e).

**Fig. 7:**
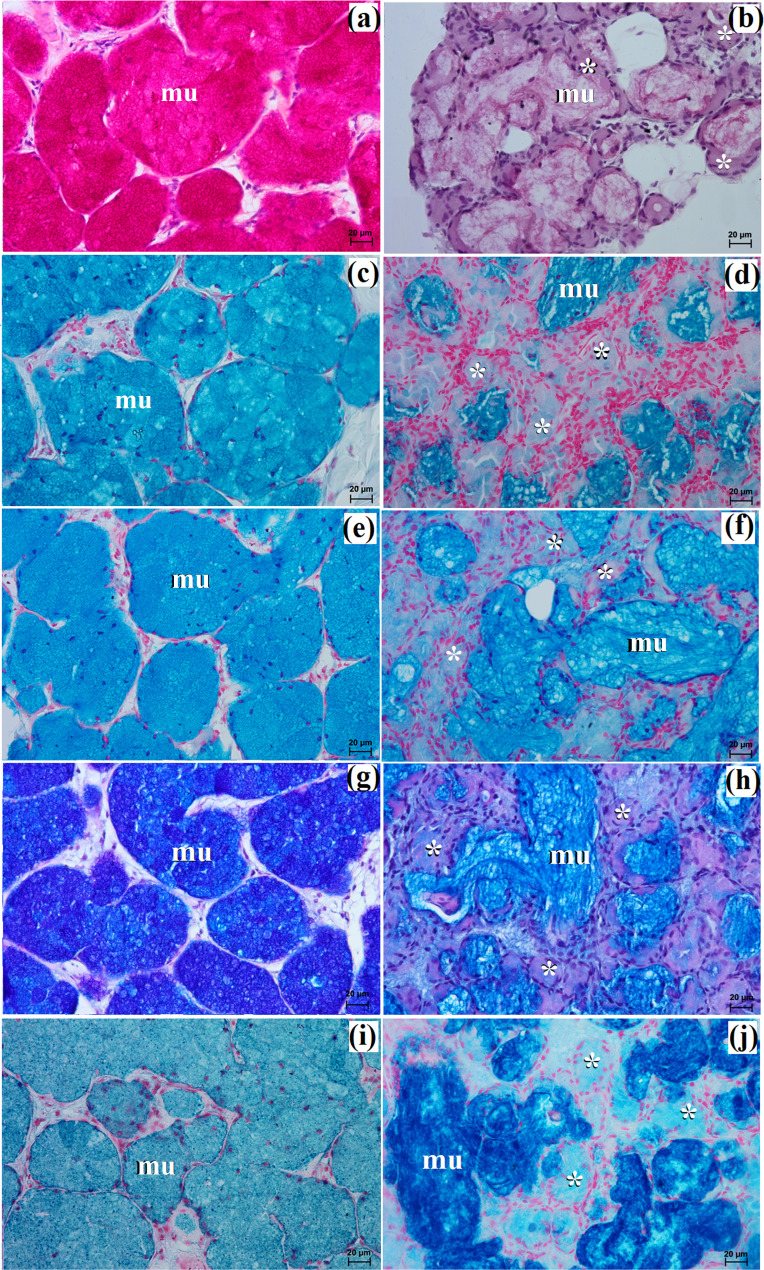
Histochemical images of the monostomatic sublingual gland in the captive South African painted dog (a, c, e, g, i) and captive fennec fox (**b**, **d**, **f**, **h**, **j**). mu – mucous units, white asterisk – serous demilunes. **a**, **b** = PAS; **c**, **d** = AB pH 1.0; **e**, **f** = AB pH 2.5; **g**, **h** = AB pH 2.5/PAS; **i**, **j** = HID

The monostomatic gland in the painted dog was a branched tubular complex gland producing a mucous secretion, while tubules were composed of low pyramidal cells with broad bases, very small lumens, and oval nuclei (Figs. 5c and 7a, c, e, g and i; Table 2). In contrast, in the fennec fox, it was a branched tubuloalveolar complex gland that produced seromucous secretion, with a predominance of serous cells (Figs. 6d and 7b, d, f, h and j; Table 2). Furthermore, in the two male fennec foxes, the presence of numerous lymphocytes was observed in both the duct system and the secretory units (Fig. 6b and c). Azan trichrome staining showed a moderate (++) positive reaction in the mucous cells in the hunting dog, while mucicarmine staining showed a negative (-) reaction in the serous demilunes and a moderate (++) positive reaction in the mucous tubules in the fennec fox (Figs. 5d and 6e and f).

### The polystomatic sublingual gland

The polystomatic sublingual gland in both species was located between the mucous membrane of the proper oral cavity, the styloglossus muscle, the genioglossus muscle, and the symphysis of the mandible (Fig. 1e and f). It was narrow and elongated in shape. It consisted of individual glandular lobules, each with its own minor sublingual ducts that opened into the lateral sublingual recess. The topographic extent of the monostomatic and polystomatic sublingual glands ranged from the mental angle to the palatoglossal arch.

**Fig. 8:**
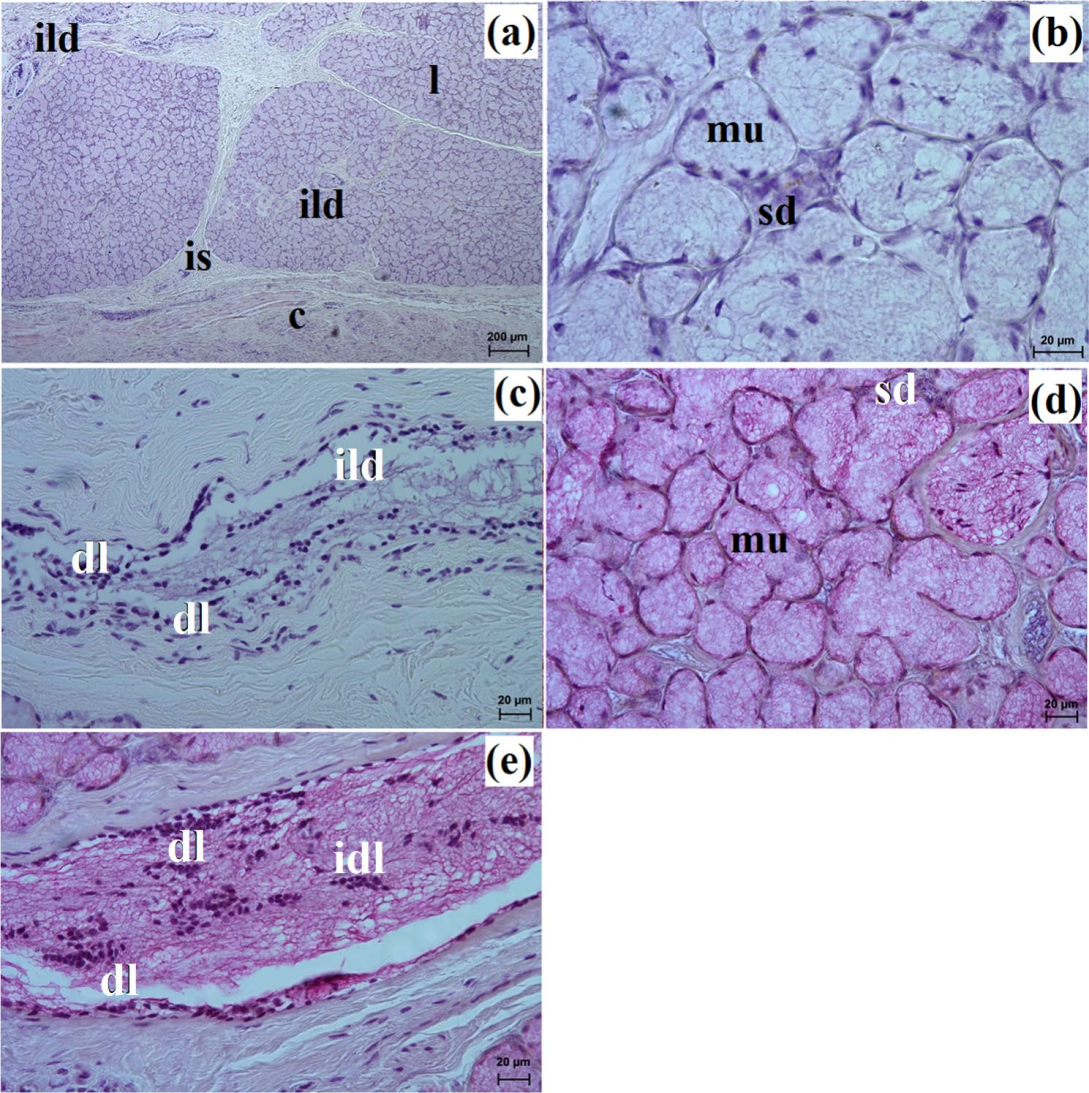
Histology images of the polystomatic sublingual gland in the captive South African painted dog. c – capsule, dl – diffuse lymphocytes, ild – intralobular duct, is – interlobar septa, l – lobes, mu – mucous units, sd – striated duct. **a** – **c** = H&E stain; **d** – **e** = mucicarmine stain

**Fig. 9:**
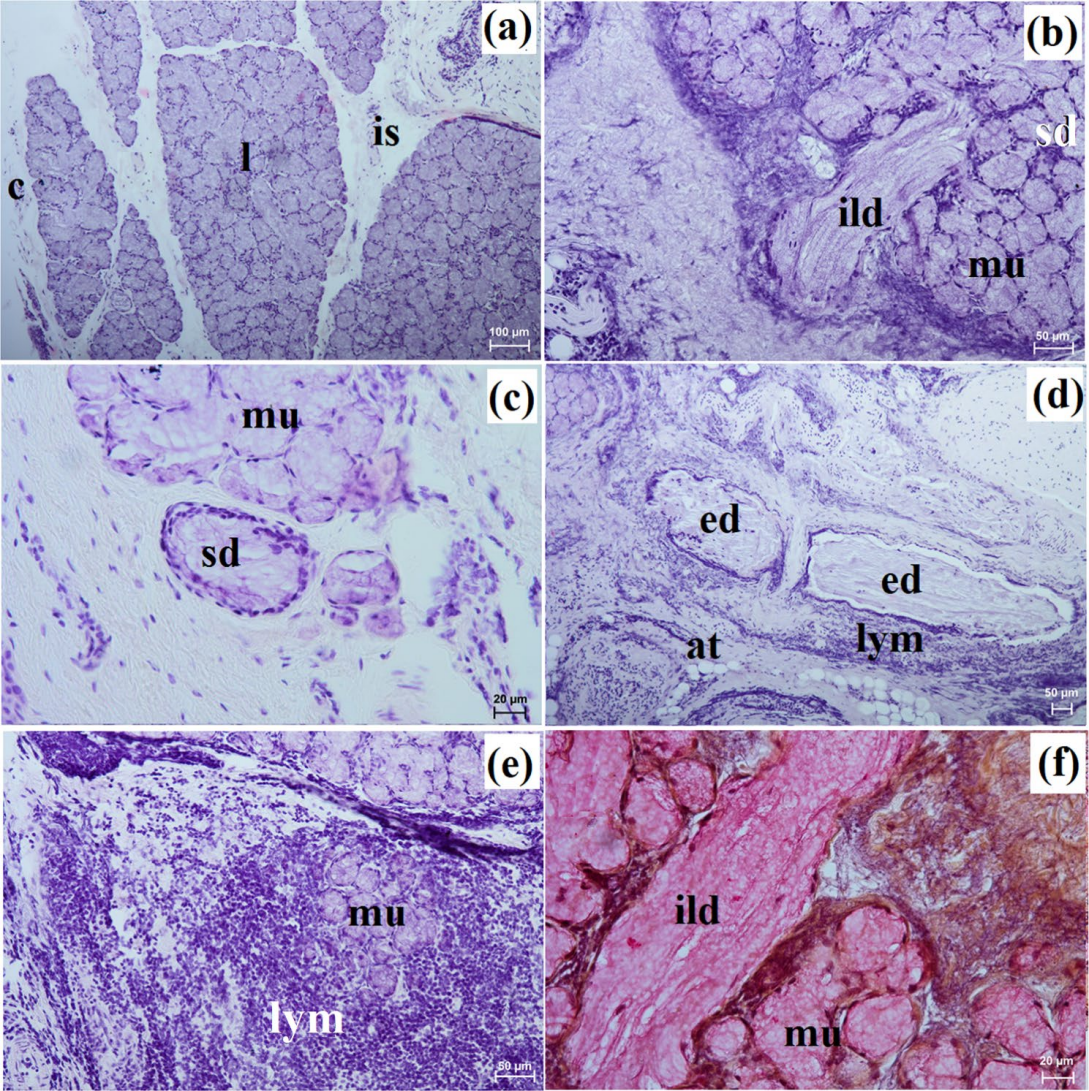
Histology images of the polystomatic sublingual gland in the captive fennec fox. at – adipose tissue, c – capsule, ed – excretory duct, ild – intralobular duct, is – interlobar septa, l – lobes, lym – lymphocytes, mu – mucous units, sd – striated duct. **a** – **e** = H&E stain; **f** = mucicarmine stain

In the South African painted dog, this gland was covered by a thick connective tissue capsule (Fig. 8a), while in the fennec fox, this capsule was thin (Fig. 9a).

**Fig. 10:**
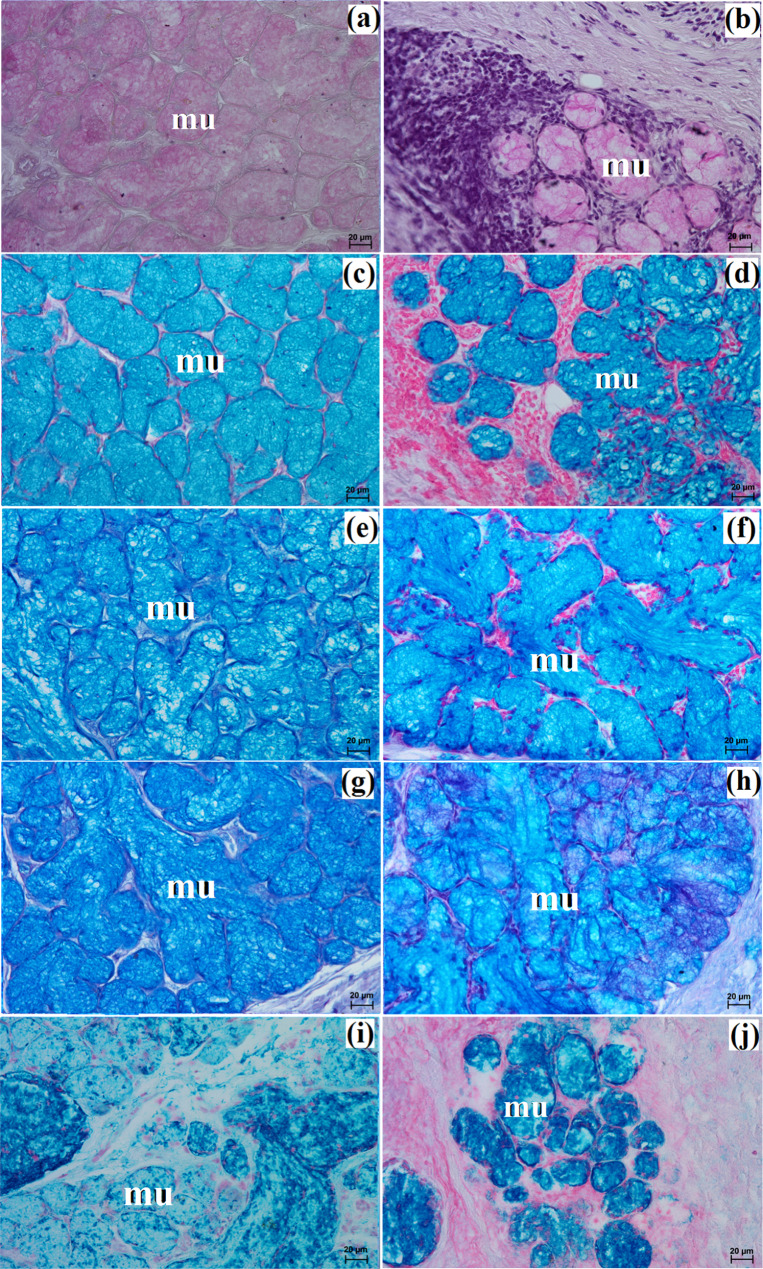
Histochemical images of the polystomatic sublingual gland in the captive South African painted dog (a, c, e, g, i) and captive fennec fox (**b**, **d**, **f**, **h**, **j**). mu – mucous units. **a**, **b** = PAS; **c**, **d** = AB pH 1.0; **e**, **f** = AB pH 2.5; **g**, **h** = AB pH 2.5/PAS; **i**, **j** = HID

In the painted dog, it was divided by thick connective tissue septa into large lobes (oval, elongated), which to a small extent underwent secondary division into smaller segments through thin septa (Fig. 8a). In the fennec fox, a visible thick septa divided the gland parenchyma into large and small lobes of various shapes (oval, elongated, triangular, and quadrilateral) without signs of secondary division (Fig. 9a). The duct system was well-developed in all animals (Figs. 8a and b and 9a and c). In both the South African painted dog and the fennec fox, the polystomatic sublingual gland was a branched tubular complex gland producing mucous secretion (Figs. 8b, 9c and 10a and j; Table 2). Furthermore, in the three female hunting dogs, numerous diffuse lymphocytes were observed around intralobular ducts, while in the two male fennec foxes, clusters of lymphocytes around striated ducts and secretory units were observed (Figs. 8c and 9d and e). Mucicarmine staining showed a strong (+++) positive reaction in mucous cells in the Cape hunting dog, while in the fennec fox, it was a moderate (++) positive reaction (Figs. 8d and e and 9f).

### The mandibular gland

The mandibular gland in the South African painted dog was located in the retromandibular fossa, bounded dorsally by the posterior edge of the ramus of the mandible, dorsally and caudally by the atlas wing, and ventrally by the basihyoid. This gland was covered by a thick connective tissue capsule. The dorsally bordered edge of the gland was overlaid by a portion of the parotid gland, while the ventral edge contacted the thyrohyoid muscle and lingual vein. The caudal edge was defined by the external jugular vein and maxillary vein, while the rostral edge made contact with a portion of the parotid gland and mandibular lymph nodes (Fig. 1g). In the painted dog, it had an elongated oval shape (Fig. 1g). In contrast, in the fennec fox, this gland was located behind the ramus of mandible, at the level of the atlas wings. It was delimited by the caudal edge of the parotid gland, the sternothyroideus muscle and the mastoid part and the occipital part of the sternocephalicus muscle (Fig. 1h). In the fox, the mandibular gland had a sherical shape. The mandibular duct in both species ran between the tongue muscles and the body of the mandible and opened into the oral cavity proper at the sublingual caruncle (Fig. 1i and j).

**Fig. 11:**
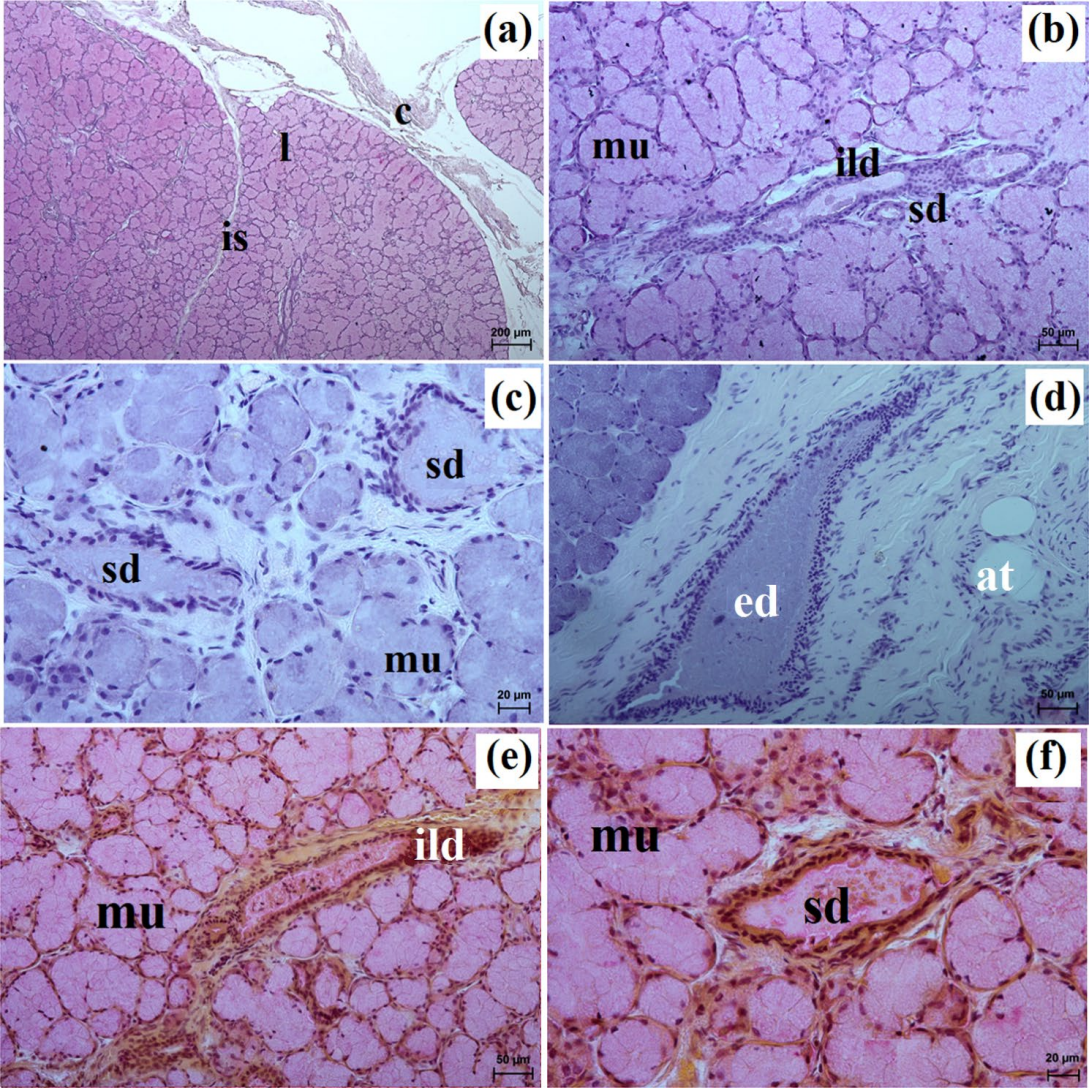
Histology images of the mandibular gland in the captive South African painted dog. at – adipose tissue, c – capsule, ed – excretory ducts, ild – intralobular duct, is – interlobar septa, l – lobes, mu – mucous units, sd – striated duct. **a** – **d** = H&E stain; **e** – **f** = mucicarmine stain

**Fig. 12:**
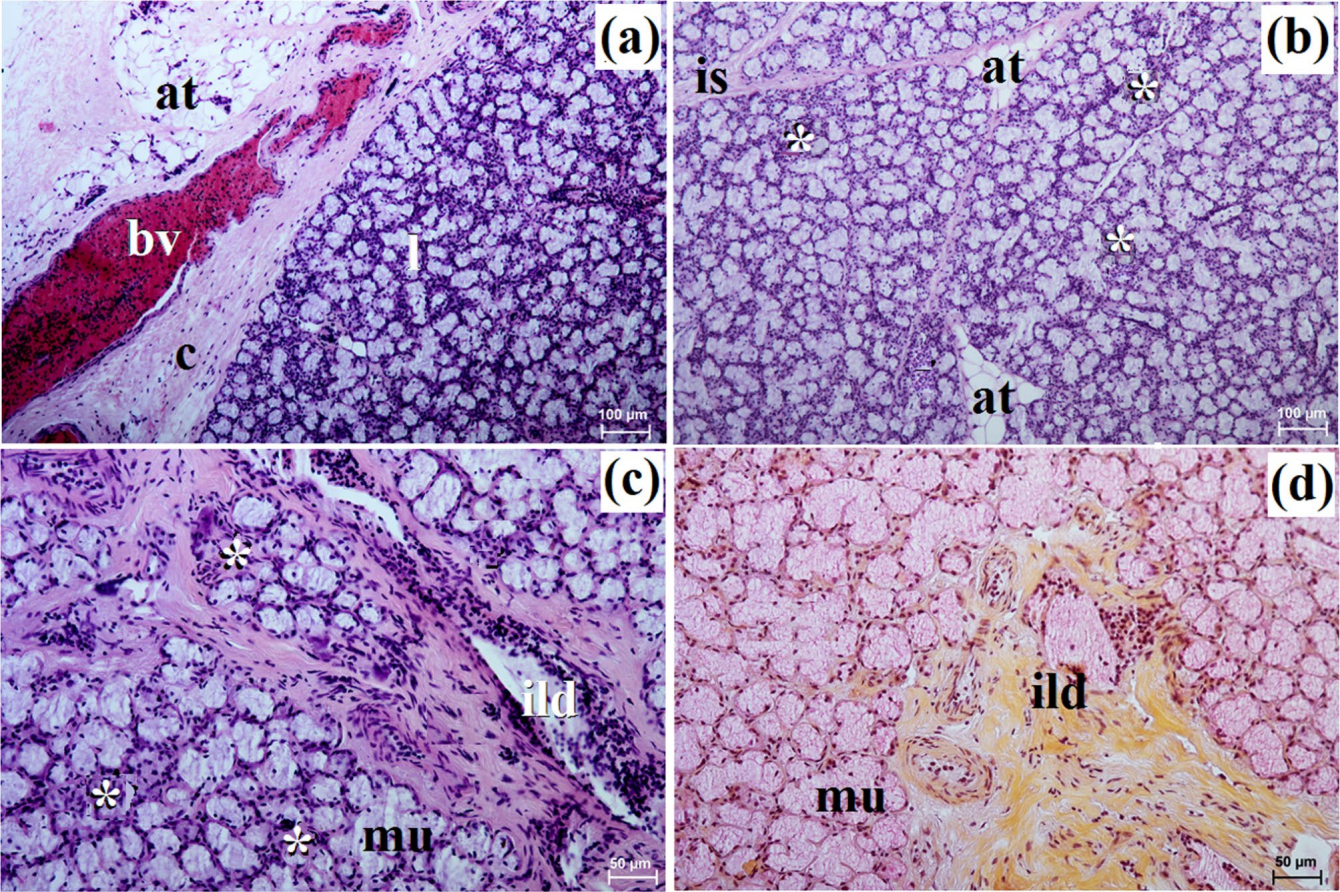
Histology images of the mandibular gland in the captive fennec fox. at – adipose tissue, bv – blood vessels, c – capsule, ild – intralobular duct, is – interlobar septa, l – lobes, mu – mucous units, white asterisk – serous demilunes. **a** – **c** = H&E stain; **d** = mucicarmine stain

In the South African painted dog and fennec fox, these glands were surrounded by a thick connective tissue capsule, which extended inwardly into the gland with both thick and thin interlobar septa, dividing the gland parenchyma into lobes of various sizes – large, medium, small (Figs. 11a and 12a).

**Fig. 13:**
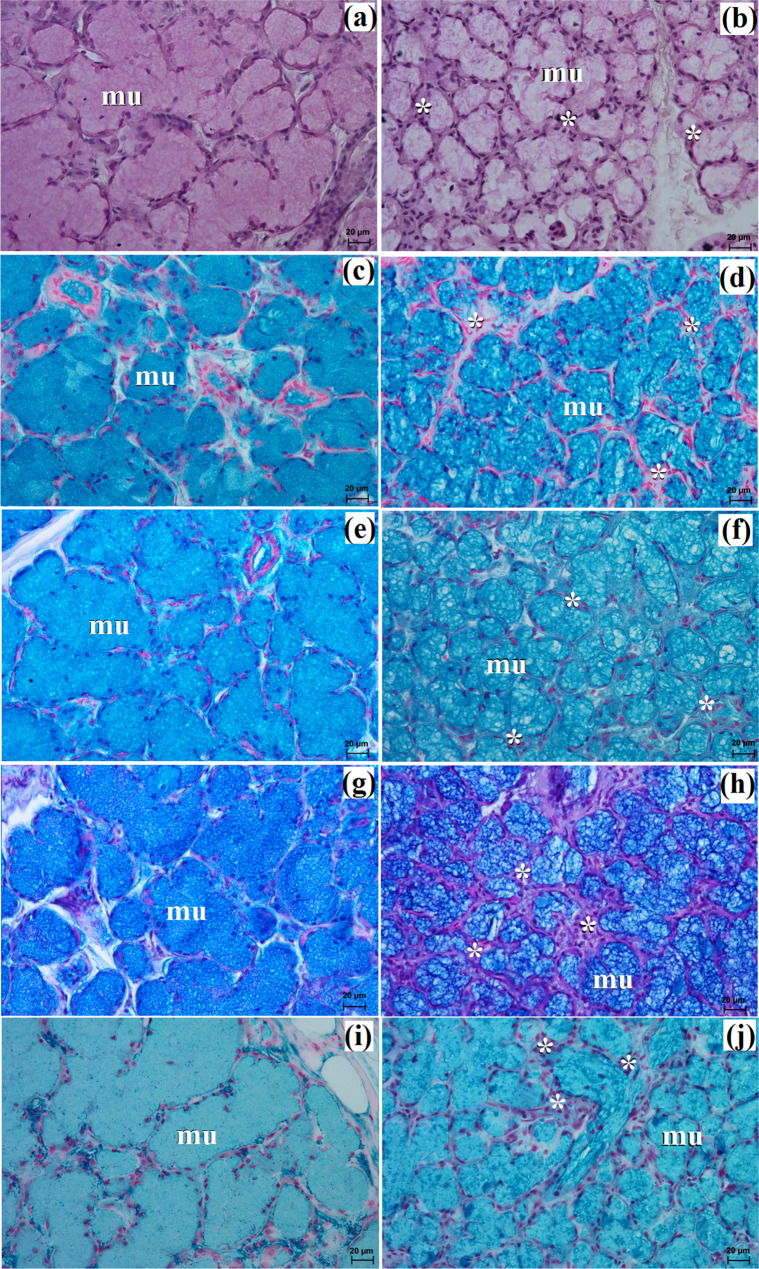
Histochemistry images of the mandibular gland in the captive South African painted dog (**a**, **c**, **e**, **g**, **i**) and captive fennec fox (**b**, **d**, **f**, **h**, **j**). mu – mucous units, white asterisk – serous demilunes. **a**, **b** = PAS; **c**, **d** = AB pH 1.0; **e**, **f** = AB pH 2.5; **g**, **h** = AB pH 2.5/PAS; **i**, **j** = HID

Large lobes predominated in size among the examined species. Furthermore, in fennec fox, the presence of clusters of adipose cells was observed within the capsule and between interlobar septa (Fig. 12a and b). The duct system in all examined individuals was well-developed (Figs. 11b and c and 12c). Furthermore, in the painted dogs, an excretory duct with broad lumina was visible, formed by simple tall columnar cells with oval nuclei (Fig. 11d). In the hunting dogs, this gland was a branched tubular complex gland producing a mucous secretion (Figs. 11c and 13a, c, e, g and i; Table 2). Meanwhile, in the fennec fox, this gland was a branched tubuloalveolar complex gland producing a mucoserous secretion (Figs. 12c and 13b, d, f, h and j; Table 2). Mucicarmine staining in the South African painted dog showed a moderate (++) positive reaction in mucous cells (Fig. 11e and f). However, a weak (+) positive reaction in the tubules and a negative (-) reaction in the serous demilunes were observed in the fennec fox (Fig. 12d).

### The parotid gland

The parotid gland in all examined carnivores was located between the ramus of the mandible and the wings of the atlas in the retromandibular fossa adjacent to the anular cartilage (external acustic meatus). Externally, it was covered by the parotid fascia and a well-developed parotidoauricularis muscle. In the South African painted dog, the parotid gland had five angles: rostrodorsal - well-developed, located near the auricle above the anular cartilage and on the frontoscutular muscle; rostral - also well-developed - contacting the buccal branch and auriculopalebral nerve (facial nerve) and masseter muscle; rostroventral- long and narrow, located beneath the masseter muscle and contacting the mandibular gland, facial vein, and body of the mandible; caudodorsal - well-developed, located behind the auricle; caudoventral - also well-marked, contacting the mandibular gland (Fig. 1k). However, in the fennec fox, it was triangular in shape and had three angles: rostral - the most developed - in the preauricular region; caudal - poorly marked, in the postauricular region adjacent to the external acustic meatus and contacting the maxillary vein; ventral – narrow, contacting the mandible lymph nodes and the lingual vein. The parotid duct ran on the surface of the masseter muscle, then passed onto the cheek, pierced it at the level of M1 – M2 teeth in the South African painted dog and M1 teeth in the fennec fox, and opened into the oral vestibule at the papilla parotis.

**Fig. 14:**
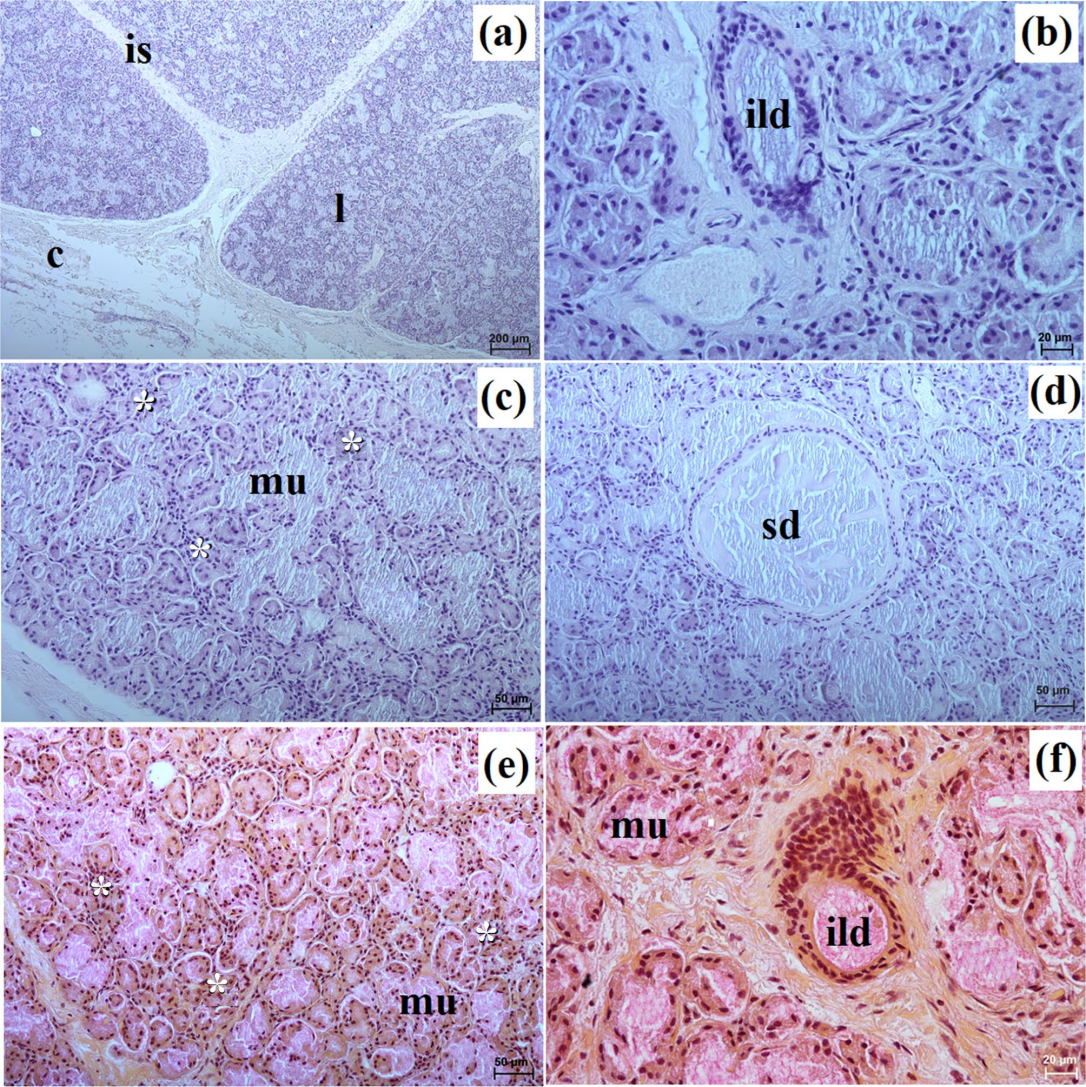
Histology images of the parotid gland in the captive South African painted dog. c – capsule, ild – intralobular duct, is – interlobar septa, l – lobes, mu – mucous units, sd – striated duct, white asterisk – serous demilunes. **a** – **d** = H&E stain; **e** – **f** = mucicarmine stain

**Fig. 15:**
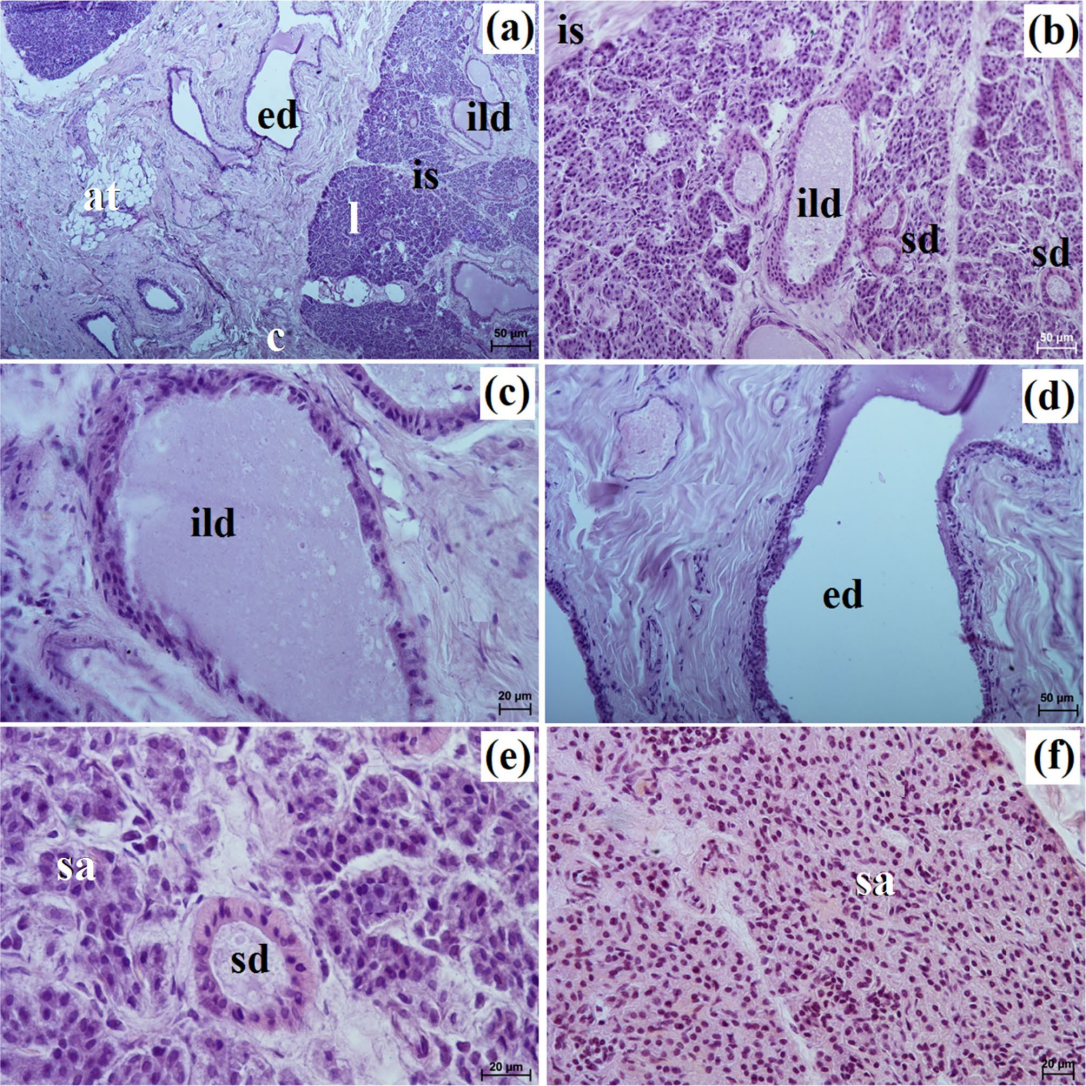
Histological images of the parotid gland in the captive fennec fox. at – adipose tissue, c – capsule, ild – intralobular duct, is – interlobar septa, l – lobes, sa– serous acini, sd – striated duct. **a** – **e** = H&E stain; **f** = mucicarmine stain

In the examined hunting dogs, the parotid gland was surrounded by a thick connective tissue capsule, while in the fennec fox, the capsule was thin (Figs. 14a and 15a).

**Fig. 16:**
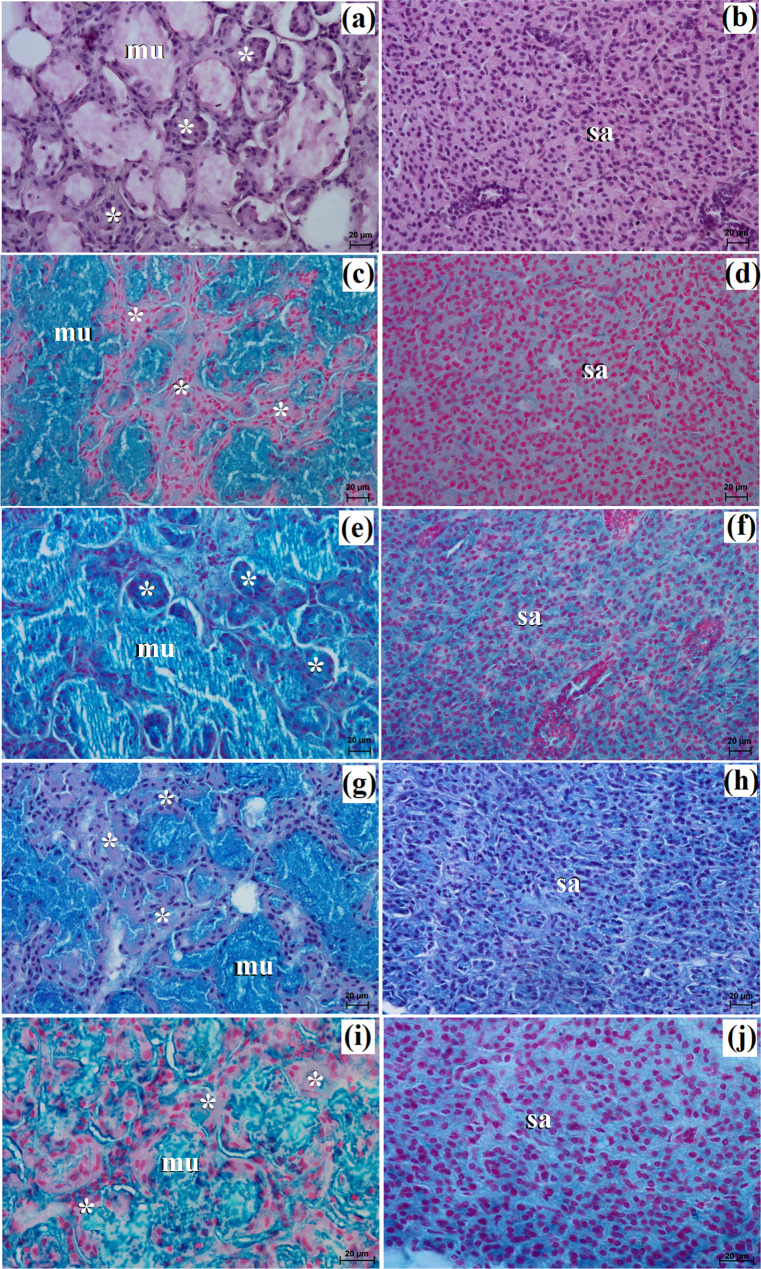
Histochemistry images of the parotid gland in the captive South African painted dog (**a**, **c**, **e**, **g**, **i**) and captive fennec fox (**b**, **d**, **f**, **h**, **j**). mu – mucous units, sa – serous acini, white asterisk – serous demilunes. **a**, **b** = PAS; **c**, **d** = AB pH 1.0; **e**, **f** = AB pH 2.5; **g**, **h** = AB pH 2.5/PAS; **i**, **j** = HID

The gland capsule in examined animals was composed of compact fibrous connective tissue, and clusters of adipose cells were visible only in the fox (Fig. 15a). Thick septa were observed in the painted dog, dividing the gland parenchyma into large lobes without secondary division features (Fig. 14a). In contrast, numerous small lobes formed by thin and thick connective tissue septa, also without secondary division, were observed in the fennec fox (Fig. 15a). The lobes in the fox had various shapes (oval, elongated, triangular, quadrilateral) (Fig. 15a), while in the hunting dog, the lobes were similar to elongated ovoid in shape (Fig. 14a). In all examined animals, numerous intralobular ducts and striated ducts were present (Figs. 14b and d and 15b and c). Furthermore, within the connective tissue of the fennec fox, an excretory duct with a broad lumina was visible, formed by simple tall columnar cells with oval nuclei located in the basal part of the cell (Fig. 15d). The parotid gland in the fox was a branched acinar complex gland producing a serous secretion (Figs. 15e and 16b, d, f, h and j; Table 2), while in the painted dog, this gland was a branched tubuloacinar complex gland producing mucoserous secretion (Figs. 14c and 16a, c, e, g and i; Table 2). Mucicarmine staining showed a negative (-) reaction in the serous acini of the fennec fox, while a moderate (++) positive reaction was observed in the tubules and a negative (-) reaction in the serous demilunes of the South African painted dog (Figs. 14e and f and 15f).

## Discussion

Food items offered to captive carnivores kept under zoological conditions are limited compared to those offered to predators in nature. Beef is the most common food source, supplemented with veal, chicken, and fish, depending on the species and individual preferences. Some zoos may occasionally offer pre-made canned dog and cat food.

To ensure a complete and balanced diet, especially when feeding a raw meat-based diet, carnivores in zoos often receive additional supplements formulated with essential vitamins and minerals [49, 50]. Small canids tend to consume a significant amount of small vertebrates, invertebrates, as well as a certain share of plant-based products (fruits and vegetables) [50].

The diet of South African painted dogs at the Wroclaw Zoological Garden is a bone-meat-based diet, supplemented with rabbits, guinea pigs, and the Mazuri D3 formula (information from the Wroclaw Zoological Garden). The diet of fennec foxes at the Wroclaw Zoological Garden included beef, mice, day-old chicks, eggs, chicken giblets, insects (such as mealworms and superworms), and some occasional fruits (information from the Wroclaw Zoological Garden).

### The zygomatic gland

The topography of the zygomatic gland in our South African painted dog corresponds to its location in the domestic dog, domestic cat (*Felis catus*), crab-eating raccoon, and ferret (*Mustela* (*putorius*) *furo*) [14, 21, 22, 33, 51–53]. However, in the fennec fox, the location of this gland is very specific: in the nasal part of the zygomatic arch, near the pterygopalatine fossa, at the border between the lacrimal and zygomatic facial parts.

Morphometric measurements among the examined individuals showed that the zygomatic gland, out of all the other examined glands, was the smallest. However, in terms of size, in the painted dog it was similar to those observed in the dog [21]. In contrast, in the fennec fox, this gland was small even compared to the domestic cat [51].

In both of our examined species, the zygomatic gland was irregular in shape, similar to the Philippine non-descript dog [54]. In other canids, it was conical in shape (domestic dog: breed dogs, mesocephalic dogs, and mongrel dogs) [21, 34, 51, 55]. In the crab-eating raccoon, it was small and rounded [52], while in the ferret, it was roughly pyramidal in shape [53].

The major duct of the zygomatic gland in the South African painted dog had 5 to 6 ducts, while in the fennec fox there were 3 to 4 ducts that opened at the height of the last upper molar (M2). An observation of 4 to 5 secretory ducts also opening at the last upper molar (M2) was made in the domestic dog, and crab-eating raccoon [33, 52]. In the ferret, this duct opened opposite the upper molar teeth (M1) [53]. Abouelela et al. [51] reported that in the domestic cat, this duct was short, similar to our examined species.

Histological and histochemical analysis showed that the zygomatic gland in our examined canids was a complex branched tubular gland that produces mucous-like secretion similar to that of the domestic dog [21, 29, 32, 54]. According to Gomi et al. [23] and Mohamed [56], in the domestic dog, this secretion was mixed, similar to that of the domestic cat, Persian leopard (*Panthera pardus saxicolor*), aardwolf (*Proteles cristata*), and ferret [51, 53, 57–59].

### The monostomatic sublingual gland

The monostomatic sublingual gland in the South African painted dog and fennec fox was located in the lateral sublingual recess of the oral cavity proper, similar to the domestic dog [21, 23, 34], crab-eating fox [38], domestic cat [3, 60], crab-eating raccoon [52], polar bear (*Ursus maritimus*) [61], and ferret [53].

The size of the monostomatic sublingual gland in the examined male and female fennec foxes was very similar, while in the South African painted dog it was large compared to the domestic dog [21]. Compared to those studied by Gaber et al. [21], this gland was smaller in the mongrel dog than in our South African painted dog, which may be due to the different body sizes of the dog tested. The largest was observed in the polar bear, where its dimensions were 60 mm x 30 mm [61].

The shape of this gland in our examined species was elongated, while in the domestic cat and ferret it was quadrilateral elongated [51, 53]. In the breed and mongrel dogs it was oval with a slightly marked indentation on the rostral edge [34]. As reported by Gaber et al. [21], in this mongrel dog, this gland resembled an elongated triangle, similar to the puma (*Puma concolor*) [62] and the brown bear (*Ursus arctos*) [63]. The oval shape was observed in the polar bear [61].

In the examined animals, similar to the domestic dog, crab-eating fox, crab-eating raccoon, coati (*Nasua narica*), ferret, and puma, the major sublingual duct ran together with the mandibular duct and entered the oral cavity proper at the sublingual caruncle [21, 38, 52, 53, 62, 64].

Histological and histochemical studies showed that this sublingual gland was a branched tubuloalveolar complex gland that produces seromucous secretion in fennec fox, similar to domestic dog and domestic cat [23, 65]. Gaber et al. [21] report that the domestic dog also had mixed secretion, as well as mucous acini and serous demilunes of nearly equal density.

In the aardwolf, the monostomatic sublingual gland produces mixed secretion, although there is no information about whether the secretion is serous or mucous in nature [57, 66]. Whereas in the ferret, it is only mucous secretion, similar to our South African painted dog [53]. The striated ducts and intralobular ducts in our species’ monostomatic lingual glands and mandibular gland were also well-developed, similar to those in the domestic dog [23].

### The polystomatic sublingual gland

The topography of the polystomatic gland in our examined animals was similar to that observed in the domestic dog, domestic cat, crab-eating fox, and crab-eating raccoon [3, 21, 38, 52, 60].

In South African painted female dogs, this salivary gland was small; meanwhile, in male and female fennec foxes, the size of this gland was identical.

In our examined species, it consisted of several independent packets, each of which had its own excretory duct leading directly to the floor of the oral cavity proper. In the brown bear, the polystomatic sublingual gland consists of glandular, reduced lobules forming a discontinuous string [63].

Histological and histochemical analysis showed that in our South African painted dog and fennec fox, it was a tubular, branched complex producing mucous secretion, while in the domestic dog, it was a complex, branched tubuloalveolar gland producing seromucous secretion [32].

### The mandibular gland

The mandibular gland in the Cape hunting dog was located in the retromandibular fossa, similar to a domestic dog [21, 22, 33, 34], Baladi dog [37], crab-eating fox [38], red fox [37], ferret [53, 67], brown bear [63], polar bear [61], puma [62], crab-eating raccoon [52, 68], and coati [64, 68]. On the contrary, in the fennec fox, it was located behind the mandible ramus in the ventral neck region, on the surface of the sternocleidomastoid muscle.

In both species, the mandibular gland was smaller than the examined parotid gland. In the South African painted dog, as well as in mongrel dogs and Baladi dogs [21, 26, 33], the dimensions of this gland were similar to those found in polar bears [61]. Similarly, in the domestic cat, the mandibular gland was also smaller than the parotid gland, which is consistent with our observations in the fennec fox [51]. According to Anderson et al. [69], in aardwolves, the mandibular gland was larger compared to other major salivary glands.

In the fennec fox, this gland had a spherical shape, similar to the domestic cat [51], ferret [53], crab-eating fox [38], crab-eating raccoon [52], brown bear [63], and polar bear [61]. In contrast, in the South African painted dog, it was elongated and oval in shape. The mandibular gland in the domestic dog was oval in shape with a slightly concave rostral margin [21, 34]. However, Nazih and El-Sherif [26] report that in the Baladi dog, this gland has an oval to elliptical outline.

The mandibular duct in our species had a similar course to that of the domestic dog, domestic cat, crab-eating fox, crab-eating raccoon, coati, puma, and ferret [2, 3, 33, 38, 51–53, 62, 64].

Histological and histochemical studies showed that the mandibular gland in the South African painted dog was a complex branched tubular gland producing mucous secretion, similar to the aardwolf [57, 66] and ferret [53, 70]. However, in the fennec fox, there was a branched tubuloalveolar system that produces mucoserous secretions, similar to the domestic dog and domestic cat [21, 23, 28, 31, 32, 51, 71, 72]. The mixed secretion of the mandibular gland was also observed in the coati [64], striped skunk (*Mephitis mephitis*), and raccoon (*Procyon lotor*) [73].

### The parotid gland

The localization of the parotid gland in our examined species was similar to other canids, such as the domestic dog [21, 22, 33, 34], Baladi dog [36], crab-eating fox [38, 39], red fox [36], and pampas fox [39]. Morphometric studies of the parotid gland in all examined animals revealed that this gland was the largest among the salivary glands, similar to observations in the crab-eating fox [38]. Interestingly, in the South African painted dog, the size of the parotid gland was proportionally similar to that in the domestic dog (weighing 10–15 kg) [21]. Due to differences in body size among breed dogs, mongrel dogs, and other canid species, the size of the parotid gland—and other salivary glands—varies among these animals.

The shape of the parotid gland in the Cape hunting dog had a clearly marked five angles, while in the fennec fox, it consisted of three angles, similar to the domestic dog, Baladi dog, red fox, and crab-eating fox [21, 33, 34, 37, 38, 51]. The parotid duct in the South African painted dog opened at the height of the first (M1) upper and second molar (M2), while in the fennec fox, it opened at the height of the first molar (M1), similarly to the maned wolf (*Chrysocyon brachyurus*) [74]. According to Souza et al. [39], in the crab-eating fox and Pampas fox, the parotid ducts opened in 53.6% of cases between the third (P3) and fourth (P4) upper premolars, and in 46.4% of cases at the level of the first upper molar (M1). On the contrary, in the Baladi dog, this duct pierced the cheek wall at the level of the four upper premolars (P4) [68]; between the third (P3) and fourth (P4) upper premolar tooth in the red fox and domestic dog [37]; and at the level of the third premolar tooth (P3) in the domestic dog [33].

The histological and histochemical study in our examined species showed that the parotid gland was a complex branched alveolar gland with serous secretion, similar to the domestic dog [21, 23, 31] and Baladi dog [37]. However, in the red fox, this gland was characterized by serous and mucous secretory units [37]. The parotid gland duct system was well-developed in all animals examined, similarly to the aforementioned species.

According to Gaber et al. [21], based on morphological studies on major salivary glands in mongrel dogs, they concluded that the nature of secretion of the salivary glands depends on the type of the dog’s diet. Dogs that are on a dry diet secrete saliva presumed to be serous, while dogs that have a meat-based diet secrete saliva that is much more mucous. In addition, they further state that the parotid gland becomes more active in dogs on a dry food-based diet, and its secretion is mainly serous, while the mandibular gland and the zygomatic gland secrete mainly mucous-nature secretion in dogs that mainly consume raw meat.

Furthermore, in three female painted dogs and two male fennec foxes, numerous lymphocytes were observed surrounfing the intralobular ducts, suggesting an ongoing inflammatory process in the monostomatic sublingual gland and the polystomatic sublingual gland.

Furthermore, in three female painted dogs and two male fennec foxes, numerous lymphocytes were observed surrounding the intralobular ducts, suggesting an ongoing inflammatory process in both the monostomatic and polystomatic sublingual glands. Based on the medical history, the inflammation in these salivary glands of our study subjects could have been triggered by mechanical trauma or underlying glandular dysfunction. The literature indicates that foreign bodies in the oral cavity are a common cause of salivary gland inflammation in dogs, as they can irritate the mucous membrane. In severe cases, this can lead to blocked ducts, resulting in saliva accumulation within the gland and the formation of an abscess. Additionally, low humidity and dental issues, such as food impaction and bacterial growth, have been associated with salivary gland inflammation in dogs [40, 41, 75].

## Conclusions

The morphology and character of secretory products of the major salivary glands (parotid, mandibular, sublingual glands - monostomatic and polystomatic, and zygomatic gland) differed between the South African painted dog (carnivore) and fennec fox (omnivore), potentially reflecting dietary adaptations.

The aforementioned studies have shown that the structure of the major salivary glands and zygomatic gland, including their arrangement, shape, and cellular composition, in both, captive South African painted dogs and fennec foxes, was comparable. Moreover, histochemical analysis of the chemical composition within examined glands revealed similar results, suggesting a shared function in all above-mentioned carnivores. This suggests that there is a common evolutionary ancestor for these canids and other terrestrial carnivores. The structures of the salivary glands have probably been conserved throughout their development. Our findings suggest that despite some dietary differences between painted dogs (carnivores) and fennec foxes (omnivores), the basic function and structure of their salivary glands remains similar to other land-based meat-eating mammals. This shared design might be particularly well-suited for processing meat-based diets.

Furthermore, this research provides valuable resources for veterinary medicine in two ways. Firstly, it serves as a guide for future treatments involving salivary glands in painted dogs and fennec foxes. Secondly, it can be treated as a baseline for identifying injury conditions within these glands, their ducts, and any abnormalities that might develop inside them.

This study of captive canids provides a strong foundation for understanding the salivary glands. It also highlights the need for further research, in order to get a more complete picture. It is recommended to analyze wild populations of South African painted dogs and fennec foxes from different populations. This would allow us to see if there are any variations in gland structure compared to their captive counterparts. Expanding the research to encompass other members of the *Vulpes* genus, like red foxes, and other canid species, such as wolves and coyotes, would be insightful. Canids exhibit remarkable dietary diversity, and studying a broader range of species could reveal how diet influences saliva composition. This, in turn, could shed light on the size, architecture, and secretory function of oral cavity glands across this fascinating group of mammals [15–19].

## Data Availability

Data is provided within the manuscript or supplementary information files.
